# Using voice to create inpatient progress notes: effects on note timeliness, quality, and physician satisfaction

**DOI:** 10.1093/jamiaopen/ooy036

**Published:** 2018-09-12

**Authors:** Thomas H Payne, W David Alonso, J Andrew Markiel, Kevin Lybarger, Ross Lordon, Meliha Yetisgen, Jennifer M Zech, Andrew A White

**Affiliations:** 1 Department of Medicine, University of Washington, Seattle, Washington, USA; 2 UW Medicine Information Technology Services, Seattle, Washington, USA; 3 Center for Scholarship in Patient Care Quality and Safety, Seattle, Washington, USA; 4 Department of Anesthesiology & Pain Medicine, University of Washington, Seattle, Washington, USA; 5 Department of Electrical Engineering, University of Washington, Seattle, Washington, USA; 6 Department of Biomedical Health Informatics and Medical Education, University of Washington, Seattle, Washington, USA

**Keywords:** electronic health records, physician documentation, automatic speech recognition, hospital care, workflow

## Abstract

**Objectives:**

We describe the evaluation of a system to create hospital progress notes using voice and electronic health record integration to determine if note timeliness, quality, and physician satisfaction are improved.

**Materials and methods:**

We conducted a randomized controlled trial to measure effects of this new method of writing inpatient progress notes, which evolved over time, on important outcomes.

**Results:**

Intervention subjects created 709 notes and control subjects created 1143 notes. When adjusting for clustering by provider and secular trends, there was no significant difference between the intervention and control groups in the time between when patients were seen on rounds and when progress notes were viewable by others (95% confidence interval −106.9 to 12.2 min). There were no significant differences in physician satisfaction or note quality between intervention and control.

**Discussion:**

Though we did not find support for the superiority of this system (Voice-Generated Enhanced Electronic Note System [VGEENS]) for our 3 primary outcomes, if notes are created using voice during or soon after rounds they are available within 10 min. Shortcomings that likely influenced subject satisfaction include the early state of our VGEENS and the short interval for system development before the randomized trial began.

**Conclusion:**

VGEENS permits voice dictation on rounds to create progress notes and can reduce delay in note availability and may reduce dependence on copy/paste within notes. Timing of dictation determines when notes are available. Capturing notes in near-real-time has potential to apply NLP and decision support sooner than when notes are typed later in the day, and to improve note accuracy.

## INTRODUCTION

Physician progress notes constitute an essential record of clinical care and facilitate communication with care team members. They also support research, measurements of care quality, automated and manual quality improvement, and billing. Gradually, more patients are engaging in their care by reading progress notes.^1^ Over the last 30 years, providers have increasingly created clinical notes using electronic health record (EHR) documentation tools. This trend accelerated markedly after the introduction of incentives for use of EHR systems in the American Recovery and Reinvestment Act of 2009.^2^ The transition from paper to electronic documentation yielded many advantages, including permitting multiple simultaneous access to notes, improved legibility, and the ability to more easily search notes.

However, electronic notes are criticized for poor readability, overuse of copy and paste, and excessive length in part due to the unfiltered importation of data stored in other parts of the EHR.^3^ Physicians have voiced concerns that writing notes in EHRs takes more time than it should^4–6^; consequently, progress notes may not be completed and available to other care team members until late in the day, impairing care coordination.^7^ Degraded clinical documentation has also contributed to widespread physician dissatisfaction with EHRs^8^ and lost productivity.^9^^,^^10^ Most concerning is the perception that electronic notes may not be accurate,^11–14^ which threatens their primary use—to aid in caring for patients—as well as key secondary uses such as research.

Though voice recognition software,^15^ scribes,^16^ and other novel documentation aids have addressed some of these concerns in clinics, the distinct inpatient workflow is less conducive to adopting these approaches during physician rounds. Unlike clinic-based physicians, inpatient physicians must move repeatedly between wards and buildings to see their patients, making mobile solutions particularly valuable. Traditional dictation turn-around-time and cost are barriers to broader use of dictation for inpatient progress notes. New documentation solutions should be developed to minimize trade-offs between efficiency, cost, timeliness, and note quality.

### Objectives

We created and tested a new approach to creating progress notes to address these concerns. In this article, we describe the evaluation of a Voice-Generated Enhanced Electronic Note System (VGEENS), integrating voice recognition with natural language processing and links to the EHR. We present results of a randomized controlled trial to measure effects of this new method of writing inpatient progress notes on note timeliness, quality, and physician satisfaction. We hypothesized that VGEENS would improve the primary outcomes compared with usual note-writing methods.

## METHODS

### Setting and system description

This work was conducted by clinicians on the inpatient general medicine services at University of Washington (UW) Medical Center and Harborview Medical Center, which are major teaching hospitals of the University of Washington with approximately 35 000 combined admissions annually. The transition from paper to electronic notes occurred in 2006 using Cerner Millennium (Cerner Corp., Kansas City, MO, USA).^17^ Prior to the work described here, nearly all progress notes on these inpatient services were typed using the Clinical Notes Editor, based on templates that automatically import patient-specific data such as medication lists, vital signs, and laboratory results.

**Figure 1.**
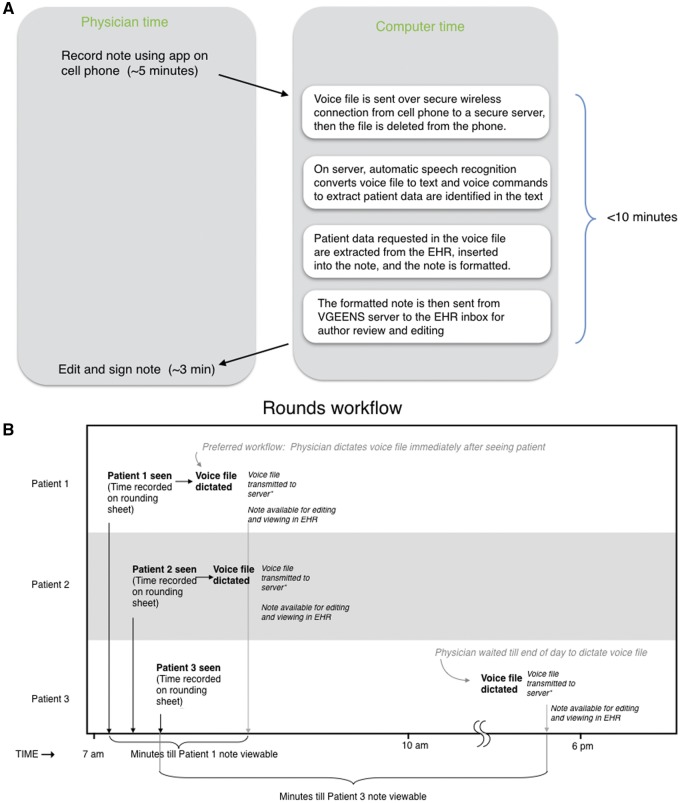
(A) VGEENS design overview. Physician records note on rounds, then voice file sent to server, converted to text, formatted, and placed into EHR inbox. (B) Rounding workflow. This figure shows the example of 3 patients seen on rounds. (Usually ∼10 patients are seen on rounds.) The physician records the time each patient is seen on the rounding sheet. The number of minutes between that time and the time the note is available to be viewed by others is a study outcome. This interval is determined by the time the voice file is dictated. If dictated immediately after seeing the patient on rounds (preferred workflow, Patients 1 and 2) it is short; if dictated at the end of the day (Patient 3), it is much longer (A).

We developed a system for physicians to create inpatient progress notes using voice (Figure 1). The system differs from commercial note writing systems available at the time of this work in its suitability to the physician rounding workflow, interaction with a commercial EHR to extract data and insert notes, and in other respects. A detailed description of VGEENS and its technical features is provided elsewhere.^18^ In brief, the VGEENS was used by physicians during or after rounds to record a voice file on a cell phone with an Android application developed by one of the authors (W.D.A.) in conjunction with the study investigators. After completing the recording, the physician pressed a “send” button causing the voice file to be securely transferred via the hospital wireless network to a server for processing and to be deleted from the phone. On the server, the file containing the digitally recorded dictation was converted to text using licensed automated speech recognition software (Dragon Medical Practice Edition, Nuance) without interactive editing, using the physician’s voice profile. Voice commands were used to break the note into sections corresponding to the preferred UW progress note format (Chief Complaint, Interval History, Exam, Laboratory and Imaging, Assessment and Plan). During the study additional voice commands to insert vital signs and laboratory results became available (eg, “Insert CBC”). The transcribed, formatted note was sent to the EHR. These coordinated automated steps occur within 10 min of the creation of the voice file on the cell phone application. From the EHR inbox, the physician could edit the document, select recipients to whom the note will be sent, and then sign it, placing it in the patient’s EHR record. The automatic note formatting and insertion of patient data in response to voice commands were introduced during the trial. Of the 709 intervention notes, 452 (64%) were produced before formatting and 497 (70%) were produced before automatic data insertion were available (The trial began December 17, 2015, automatic formatting was introduced April 17, 2016, automatic data insertion was introduced May 19, 2016, and the study ended August 30, 2016.).

All cell phone features other than Wi-Fi were disabled and the system was reviewed and approved by the UW Medicine security team.

### Study design and participants

**Figure 2.**
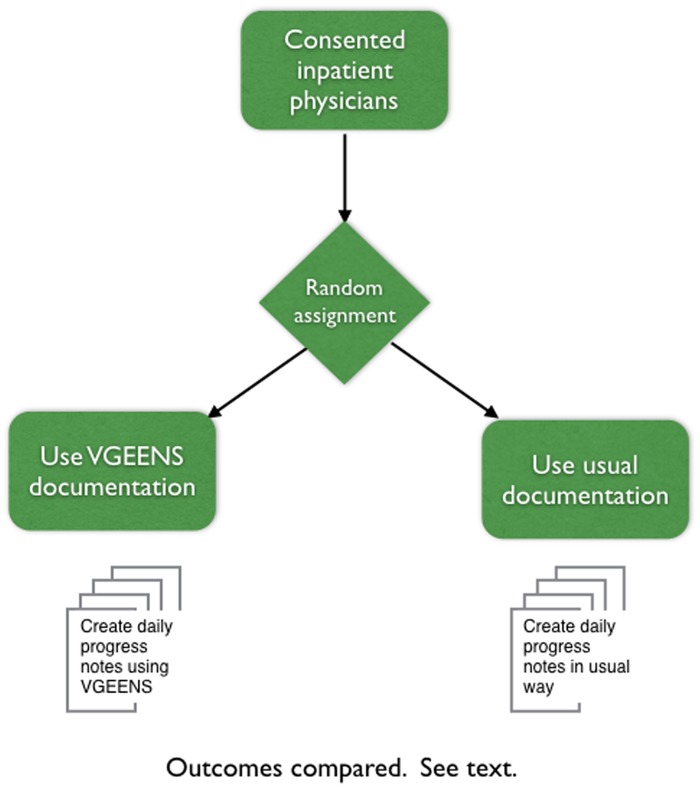
Design of randomized controlled trial to measure effect of VGEENS on study outcomes.

To test the effect of VGEENS, we conducted a randomized controlled trial between December 17, 2015 and August 30, 2016. All internal medicine residents and attending hospitalist physicians at the study sites were contacted through meetings and email messages and invited to participate in a trial of VGEENS. After a description of the study, physicians who consented to participate were randomly assigned to either the intervention or control group (Figure 2). The control group created progress notes as they usually do, typing notes using a locally developed template in Cerner’s Clinical Note Editor. The intervention group received a 20 min in-person orientation to VGEENS, were asked to train their Dragon speech profile, and used VGEENS to create progress notes as described above. Because some participants who were randomized were not on a medical service rotation in which their responsibilities included writing daily progress notes during the study period they did not contribute notes. This situation was more common in the intervention group than in the control group. There were 13 intervention subjects and 18 control subjects who contributed at least one note to the study. The UW Institutional Review Board approved this study.

### Outcomes

We compared intervention and control notes using 3 outcomes. (1) Note timeliness: the difference between the time the subject completed the patient visit during hospital rounds and the time the electronic progress note was available in the EHR for authorized users to view. (2) Physician satisfaction: satisfaction with the process of creating notes was measured using a modification of the Canada Health Infoway System and Use Assessment Survey.^19^ The 3 survey outcomes covering overall user satisfaction, system quality, and information quality were adapted for use in this study. (3) Note quality: note quality was assessed using the PQRI-9^20^ survey and a single question: “Please rate the overall quality of this note’ with a 5-point Likert scale from 1 (‘very poor’) to 5 (‘excellent’).”

### Data collection

#### Note timing

Both intervention and control physicians recorded the time they finished seeing each patient during morning rounds, documenting data on a paper sheet for each day they participated in the study. If during rounds they saw 10 patients, then the sheet would list the time they visited each patient, next to the name of each patient. For example, the rounding sheet might show Jones 7:35; Smith 8:00 am, and so on, with 10 entries. The sheets were placed in a box in a secure patient care area and collected by study personnel. The number of times recorded each day indicated the physician’s hospital census for that day. Electronic progress note metadata (but not the text of the note) including EHR logs showing when the notes were created, when they were viewable in the EHR, and when they were signed, were obtained from our analytical data repository (Enterprise Data Warehouse, Caradigm) which contains a subset of EHR data extracted for research. We determined the number of minutes between the patient visit and the availability of a viewable progress note in the EHR by subtracting the time the patient was seen on rounds from the time the note was viewable (intervention notes are viewable by others when transcribed; control notes are viewable when signed). We excluded all note types other than progress notes (eg, discharge summaries), patients for whom there was a note but no rounding time recorded, and timing data on patients who did not have a progress note written by a consented participating physician.

#### Physician satisfaction

Physicians were asked to complete a 9-item questionnaire at the conclusion of the trial. The survey used Likert scale responses to rate the system they used to write notes, evaluating satisfaction, productivity, job ease, acceptability, and support of care quality, communication, and information sharing. A short response section allowed additional comments about their experience. The survey was distributed by email and administered electronically using survey tools implemented in the UW Catalyst toolkit.

#### Note quality

We recruited 3 attending physicians who had not participated in the study to assess note quality. Notes were de-identified using an automated de-identification software package.^21^ Intervention and control notes were randomly interspersed in the set of notes given to the quality assessors, and all formatting that might give an indication of intervention or control status was stripped. Each reviewer scored a randomly selected and randomly sorted subset of 50 intervention and 50 control notes. Reviewers were blinded to the note creation method. Half of the notes (100) were evaluated by 2 reviewers. Reviewers received a $25 card to compensate them for the time spent doing this review. Reviewers assigned scores for all domains of the PQRI-9 instrument and an overall quality score. The PQRI-9 scores from the 2 reviewers who assigned scores to the same notes were averaged for analysis.

### Statistical analysis

Differences between intervention and control groups in note availability delay were compared using a random effects general least squares regression to determine if there were clustering by provider or secular trends. Outcomes were assessed using intention-to-treat approach^22^ (we combined outcomes in notes that intervention subjects created both with VGEENS and notes they created by typing) in subjects who contributed at least one note. Physician survey responses regarding satisfaction were dichotomized to combine “Highly satisfied” and “satisfied” versus all others and compared using χ^2^. In analysis of note quality, we used proportional weighting in calculating descriptive statistics such as means because the number of codes assigned to notes differed between notes. To test our hypotheses around differences between the intervention and control groups across these scores, we needed to take into account not only these proportional weights, but also clustering by note author, as individual physicians contributed anywhere from 1 to 78 chart notes to the sample and multiple notes authored by the same physician might be expected to have scores more similar to one another than to notes authored by a different physician. Therefore, the *P*-values in results come from mixed effects regression models, with both the chart note ID and the author ID as random effects in the model and proportional weights to take into account the differing number of ratings per chart note. The “sum of all axes” outcome was a mixed effects linear regression model; all others were mixed effects ordered logistic regression models, taking into account the fact that the individual Likert scale items must be considered as ordinal rather than a continuous outcome. Covariates in each regression model include intervention group as the primary predictor (binary), and date as a continuous variable to adjust for the potential of secular trends over time. As the regression analyses were based on 100 coded notes from each group and each had a date recorded, there were no missing or incomplete data to address in the regression analyses. All statistics were performed in STATA 15.0 (StataCorp LLC, College Station, TX, USA). We summarized emerging themes and suggestions for system improvement from the survey written comments.

## RESULTS

Forty-nine subjects agreed to participate (Table 1). Of these 49 subjects, 24 (49%) were randomized to intervention and 25 (51%) to the control group. Of these, 31 contributed at least one note during the study period; 18 (58%) of those were attending physicians and the others were resident physicians. Eighteen physicians did not contribute at least one note because after randomization they were not on a medical service rotation in which their responsibilities included writing daily progress notes during the study period.

**Table 1.** Characteristics of VGEENS randomized controlled trial participants

	Intervention	Control	Total
Contributed ≥1 note to study (%)			
Attending	9 (69)	9 (50)	
Female	7 (54)	14 (78)	
Subtotal	13	18	31
Did not contribute note to study (%)			
Attending	5 (55)	2 (29)	
Female	7 (64)	5 (71)	
Subtotal	11	7	18
Total consented subjects	24	25	49

*Abbreviation:* VGEENS: Voice-Generated Enhanced Electronic Note System.

**Table 2.** Outcomes of randomized controlled trial of VGEENS on note timeliness and provider satisfaction

	Intervention	Control
Rounding time recorded (%)	99	99
Notes written	709	1143
Time between rounds visit and note available to view, median (min)	227	190
Provider satisfaction with note writing process		
Respondents (% of eligible)	13 (100)	18 (100)
Satisfied, highly or moderately (%)	5 (38)	10 (56)

*Note:* Rounding time shows the percentage of patient seen on rounds where the time of rounding was recorded.

*Abbreviation:* VGEENS: Voice-Generated Enhanced Electronic Note System.

Subjects created 1852 inpatient progress notes during the study period. Of these, 1143 notes (62%) were created by control subjects and 709 notes by intervention subjects. Most of the notes (86%) were written by attending physicians. Of the notes written by intervention subjects, 499 (70.4%) were dictated using the VGEENS application and the remainder were typed, because the physician elected to type a note rather than dictate using VGEENS or because VGEENS was not operating during the 2.5% of the trial affected by unscheduled system downtime.^18^ The mean notes per user in the intervention arm was 54.5 (range 1–321, standard deviation [SD] 84.8) and in the control arm 63.5 (range 6–188, SD 54.8).

### Outcomes

#### Timeliness of note availability

**Figure 3.**
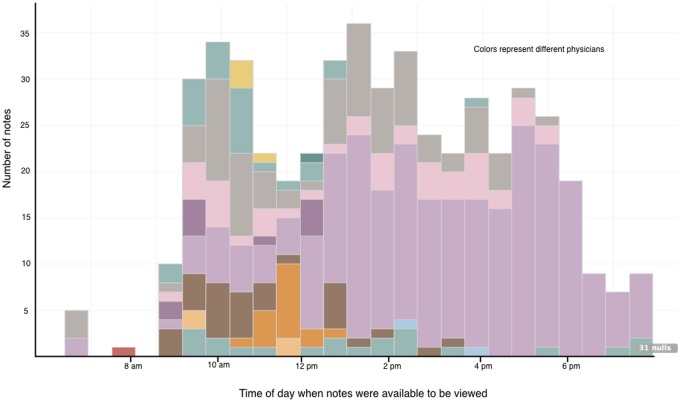
Time when intervention notes were available to be viewed.

Outcomes are summarized in (Table 2). The 31 subjects recorded rounding timing data (when patients were seen on rounds) on 1850 (99.9%) patient encounters on rounds (Figure 3). The mean time that patients were seen on rounds was 9:58 am (median 9:40 am, earliest 1:30 am, latest 6:20 pm). The median number of minutes between the patient visit on rounds and the availability of a progress note in the EHR for others to view was 190 min for the control group (mean 228, range 0–1149) and 227 min for the intervention group (mean 307, range 7–1425), an unadjusted difference of 37 min longer for intervention compared with control. When adjusting for clustering by provider and secular trends, there was no significant difference between the intervention and control groups in the time between when patients were seen on rounds and when progress notes were viewable by others (95% confidence interval −106.9 to 12.2 min).

Among the 499 notes dictated using VGEENS, the median number of minutes between the patient being seen on rounds and the availability of a progress note in the EHR for others to view was 198 min (mean 238, range 5–1420) compared with 350 (mean 472, range 7–1425) for notes that were typed (This analysis excludes notes written by intervention subjects who wrote notes without using VGEENS, eg, if VGEENS was not available or if they chose to type a note for other reasons.).

#### Satisfaction with the note writing process

*Quantitative analysis.* Forty-five of 49 subjects completed the survey, an overall response rate of 91%. We excluded from survey analysis the 18 subjects who consented to the study but did not write at least one note. The response rate for the 31 subjects who completed at least one note was 100%. Among the 13 intervention subjects, 5 (38%) reported they were either highly or moderately satisfied. Among the 18 control subjects, 10 (56%) of subjects rated their satisfaction with note writing as either highly or moderately satisfied.^10^ There was no difference between intervention and control for portion highly or moderately satisfied (*P* = .35).

*Qualitative analysis.* Survey comments illuminated subjects’ reasons for their reported satisfaction and included suggestions to improve VGEENS. Comments centered around 3 themes.

The first theme was workflow. Use of VGEENS involved a change in the workflow of creating notes. “It just really doesn't work well with resident workflow, especially the days the residents on medicine are alone. It is so much more important for progression of care for interns to get consults called during rounds, than to get notes written between patients.” “Typing out my plan helps me process easier what I want to do for my patient and think through billing. I wonder if I were further out from residency, if dictating would be as easy since I will have a better handle on things by then.”

The second theme was comments pertaining to use of voice recognition software. “I found the language processing to be typical of Dragon standards, which is to say sometimes less than stellar.” “Often doesn't understand what I'm saying.” Voice recognition errors added time to edit notes, and the requirement to format the note early in the trial before note formatting enhancements were available required editing that was also time-consuming. Some subjects, primarily resident physicians, said they did not have prior experience with dictation and so preferred using a keyboard to write notes; late in their internship year they felt they had mastered use of keyboard methods to write notes and were reluctant to learn a new technique.

The third theme was requested enhancements. “It would be helpful if it could template for you rather than having to verbally add headings for each section of the note. It would be more helpful to have this for admission or discharge notes rather than (or in addition to) progress notes, as these are typically more verbose.” These included requests that elements of the note that change less from 1 day to the next (chief complaint, problem list, and key elements of plan) should not have to be dictated anew each day and that VGEENS be available for use to create admission notes and discharge summaries. Some physicians felt creating a running summary of each patient problem with diagnostic test results was useful to prepare discharge summaries, but if notes were dictated this summary would have to be re-dictated or copied and pasted into the transcribed note.

#### Note quality

When taking into account clustering by provider and secular trends, there was no significant difference between the intervention and control groups in the overall assessment of note quality, the sum of all PQRI-9 domains, or any of the individual domains (Table 3).

**Table 3.** Note quality ratings by PQRI-9 domains, summary of domains, and overall note quality for 100 intervention and 100 control notes from the VGEENS trial

	Intervention	Control	*P*-value	Effect size^a^	*z*-score
Overall note quality	3.82 (3.67–3.98)	3.88 (3.71–4.04)	.44	0.84 (0.55–1.30)	−0.77
Up to date	3.47 (3.34–3.60)	3.48 (3.36–3.61)	.72	0.93 (0.63–1.38)	−0.36
Accurate	3.52 (3.39–3.64)	3.52 (3.39–3.66)	.85	0.97 (0.69–1.35)	−0.19
Thorough	4.04 (3.87–4.20)	3.89 (3.70–4.08)	.86	0.93 (0.43–2.03)	−0.18
Useful	4.14 (4.00–4.28)	4.16 (4.01–4.30)	.77	0.93 (0.59–1.48)	−0.29
Organized	4.03 (3.84–4.21)	4.10 (3.92–4.28)	.63	0.89 (0.57–1.40)	−0.49
Comprehensible	4.03 (3.86–4.20)	4.08 (3.90–4.25)	.53	0.87 (0.56–1.35)	−0.63
Succinct	3.71 (3.50–3.92)	4.17 (4.01–4.33)	.38	0.73 (0.36–1.47)	−0.88
Synthesized	4.23 (4.08–4.38)	4.06 (3.88–4.23)	.28	1.29 (0.81–2.07)	1.07
Internally consistent	3.67 (3.53–3.81)	3.70 (3.55–3.85)	.54	0.88 (0.59–1.32)	−0.61
Sum of all axes	34.82	35.17	.64	Beta coefficient −0.35 (−1.84 to 1.14)	−0.46

*Note:* 95% confidence interval appears in parentheses.

*Abbreviation:* VGEENS: Voice-Generated Enhanced Electronic Note System.

a Effect sizes reported as odds ratios except as indicated.

## DISCUSSION

We developed and integrated into hospital workflow a new way to create hospital physician progress notes, and evaluated it using a randomized controlled trial. Our motivation was that portable voice recognition, augmented by automated note processing, has potential to address several weaknesses of electronic notes entered by keyboard and mouse: notes take too long to create, require use of a fixed workstation, and may be less trustworthy as a result of overuse of copy/paste and the delay between rounds and when progress notes are written. We intended for this innovation to address aspects of physicians’ dissatisfaction with EHR documentation burdens.^7^

When physicians used the VGEENS method while on rounds (visible in Figure 3 as notes transcribed between 8 am and noon), notes were available for others to view within 10 min, early in the workday. The notes were automatically formatted, included patient results at the voice command of the physician, and note was integrated with a commercial EHR. This version of the VGEENS began each day’s note anew with the voice dictation and did not carry forward information such as problem list and ‘checklist’ information, though these features could be added. We leveraged a commercial EHR by using mechanisms the EHR vendor provides to extract patient data and to insert notes using the application programming interface (API) used by transcription services which is available in most EHRs. In the future, we might use FHIR^23^ for the same purposes, making this application more portable across commercial EHRs. Extending commercial EHR functionality by utilizing a growing set of APIs that vendors provide may provide functions useful to some providers.^24^

Figure 3 also demonstrates that more intervention notes were created by 1 physician than by others, and that this physician used VGEENS later in the day than most other intervention physicians. We adjusted for this as described in Statistical Analysis section. For the other physicians, notes were available earlier in the day because they used VGEENS earlier in the day. Because this single subject contributed more notes than the others, and used VGEENS later in the day, it had the effect of extending the time when interventions notes were available compared with control subjects. The figure demonstrates the potential for VGEENS to generate notes available earlier in the day if it is used on rounds as we intended.

We did not find support for the superiority of VGEENS for our 3 primary outcomes. Some physicians, especially house staff, preferred using familiar methods to create notes and did not consistently adhere to the study protocol. Some subjects reverted to typing notes at the end of the day even given the option of using voice to create them after rounds. This is not surprising—resistance to change occurred when moving from paper to electronic notes over a decade ago.^25^ It is difficult to introduce new tools that alter the complex workflow of hospital care, and resident physicians may have less control over team workflow than attending physicians. Our choice of a randomized controlled trial lowers likelihood that provider preference or technical aptitude influenced our results.

We have encouraging evidence in support of our original motivations: If progress notes are created using voice during or soon after rounds they are available to others much sooner—within 10 min. With practice, clinicians can use voice to create notes just as they use voice during their day to communicate with patients and consultants. Some of these shortcomings that likely influenced subject satisfaction are very likely due to the early state of our VGEENS and the short interval for system development before the randomized trial began, and because the study sites did not routinely offer human transcription, making dictation generally less familiar to many physicians. By the time we introduced note enhancements features to format notes and respond to voice commands to include patient data, over two-thirds of the trial was completed, so most study participants did not benefit from these features, and this likely detracted from their satisfaction with the new system.

Our work has important strengths. First, the system we developed is simple, portable, open source, uses widely available automatic speech recognition tools, requires little training, and works with a common commercial EHR. These features improve generalizability to diverse healthcare settings. Second, we evaluated use in a busy patient care setting with robust study design using a randomized controlled trial, ensuring adequate power, and limiting confounders. Third, we achieved a very high survey response rate, limiting non-response bias. Lastly, we selected important, balanced outcome measures. For example, had the intervention focused only on note timing, it might unintentionally encourage the rapid creation of low quality notes, or degrade physician satisfaction. Successful innovation in documentation should sustain or improve all 3 measures.

This study also had important limitations. Our study subjects only included physicians from 1 specialty, practicing in an academic setting, which may limit generalizability. Note quality is difficult to assess using the best available instruments, and is vulnerable to bias. We did not fulfill our intention to leverage advanced NLP techniques to correct semantic errors within the note, and to extract encoded concepts from the narrative text.^26^ These, and other refinements might have increased physician satisfaction with the system, but were not feasible in this brief demonstration project. Some of the note formatting features were introduced in mid-trial, so their use was not randomly distributed between the intervention and control groups at the beginning of the trial. The intervention was introduced to a group without pre-existing access to workstation-based voice recognition services, which may have limited adoption. Our results must be interpreted with the understanding that we both developed VGEENS and evaluated it. Self-evaluation can introduce bias that might be avoided by an independent evaluation of the system.

Perhaps the greatest promise for this work is that we have developed a system to create notes that captures physician thinking as close to rounds as possible; we have the potential to use note content to suggest diagnostic and therapeutic interventions based on that thinking in near-real-time. The VGEENS approach has potential to directly address physician concerns with excessive documentation time requirements and declining note quality and may also improve progress note accuracy. It offers an alternative but does not replace existing methods to enter progress notes. We believe use of voice will grow as a method of documenting care because it is familiar to everyone. Additional clinician training and more detailed Training voice files may improve performance and ease of use.

### Further research

We plan improvements and a new system (VIBE) and will allow use of VIBE for admission notes and discharge summaries which will permit those less familiar with dictation to gain more experience. VIBE will permit importing of annotated problem lists and daily “checklist” (eg, code status, next of kin contact information) some physicians prefer to include in notes to avoid requirement they be re-dictated daily or copied from previous notes. Another weakness of VGEENS to be addressed in the future is the cognitive burden placed on the note author to remember all important sections of the note to cover. Templates reduce this burden by including prompts and automatically inserting headers to remind the note author to be complete when creating the note. VIBE will be based on a cloud-based speech recognition engine but will retain connection to the commercial EHR to extract patient data in response to voice command and insert the completed note into the inbox. Planned and current research examines duplication of history and physical examination findings and their accuracy compared with a gold standard. We have more technical enhancements to the VGEENS underway. We know that professional note appearance is important to physicians,^27^ and so further enhancements in note formatting and to reduce editing burden may increase physician satisfaction. There are tools available in our EHR to use NLP to extract problem list elements from any note. In a study underway we are quantifying the amount of copy/paste in progress notes and will use this in future research on use of voice to create notes. Note writing workflow using VIBE permits capturing information gathered from the bedside (history and exam) immediately but also allows thoughtful additions to the assessment after rounds—the note can be edited before signing even though the bulk of the note was composed using VIBE. We plan also to measure aptitude and skill using voice and keyboard entry in future studies.

## CONCLUSION

We have developed and evaluated a system to permit physicians to create progress notes in a commercial EHR using voice using a simple approach that fits rounding workflow. Notes created are available in the EHR within 10 min.

VGEENS permits voice dictation on rounds to create progress notes and can improve note availability and may reduce dependence on copy/paste within notes. Timing of dictation determines when notes are available; in this early trial many notes were dictated long after rounds, delaying note availability. Capturing notes in near-real-time has potential to apply NLP and decision support sooner than when notes are typed later in the day, and to improve note accuracy.

## References

[1] Prey JE, Woollen J, Wilcox L, et al Patient engagement in the inpatient setting: a systematic review. J Am Med Inform Assoc 2014; 21 4: 742–50. 24272163 10.1136/amiajnl-2013-002141 PMC4078275

[2] Blumenthal D. Launching HITECH. N Engl J Med 2010; 362 5: 382–5. 20042745 10.1056/NEJMp0912825

[3] Weis JM, Levy PC. Copy, paste, and cloned notes in electronic health records: prevalence, benefits, risks, and best practice recommendations. Chest 2014; 145 3: 632–8. 10.1378/chest.13-0886 24590024

[4] Sinsky C, Colligan L, Ling LL, et al Allocation of physician time in ambulatory practice: a time and motion study in 4 specialties. Ann Intern Med 2016; 165 11: 753–60. 27595430 10.7326/M16-0961

[5] Sulmasy LS, López AM, Horwitch CA. Ethical implications of the electronic health record: in the service of the patient. J Gen Intern Med 2017; 32 8: 935–9. 28321550 10.1007/s11606-017-4030-1 PMC5515784

[6] Christino MA, Matson AP, Fischer SA, et al Paperwork versus patient care: a nationwide survey of residents' perceptions of clinical documentation requirements and patient care. J Grad Med Educ 2013; 5 4: 600–4. 24455008 10.4300/JGME-D-12-00377.1 PMC3886458

[7] Hirschtick RE. John Lennon’s elbow. JAMA 2012; 308 5: 463–4. 22851112 10.1001/jama.2012.8331

[8] Friedberg MW, Chen PG, Van Busum KR, et al RAND RESEARCH REPORT: Factors Affecting Physician Professional Satisfaction and Their Implications for Patient Care, Health Systems, and Health Policy. 2013 http://www.rand.org/pdfrd/pubs/research_reports/RR439.html (Accessed December 22, 2017). 10.7249/rhq-03-04-01 PMC5051918 28083306

[9] Lam JG, Lee BS, Chen PP. The effect of electronic health records adoption on patient visit volume at an academic ophthalmology department. BMC Health Serv Res 2015; 16 1: 7. 10.1186/s12913-015-1255-8 PMC4712610 26762304

[10] Payne TH, Corley S, Cullen TA, et al Report of the AMIA EHR 2020 task force on the status and future direction of EHRs. J Am Med Inform Assoc 2015; 22 5: 1102–10. 26024883 10.1093/jamia/ocv066 PMC5009932

[11] Hammond KW, Helbig ST, Benson CC, et al Are electronic medical records trustworthy? Observations on copying, pasting and duplication. AMIA Annu Symp Proc 2003; 2003: 269–73. PMC1480345 14728176

[12] Turchin A, Goldberg SI, Breydo E, et al Copy/paste documentation of lifestyle counseling and glycemic control in patients with diabetes: true to form? Arch Intern Med 2011; 171 15: 1393–4. 21606091 10.1001/archinternmed.2011.219 PMC3711116

[13] Hersh WR, Weiner MG, Embi PJ, et al Caveats for the use of operational electronic health record data in comparative effectiveness research. Med Care 2013; 51 (8 Suppl 3): S30–7. 23774517 10.1097/MLR.0b013e31829b1dbd PMC3748381

[14] Yadav S, Kazanji N, K C N, Paudel S, et al Comparison of accuracy of physical examination findings in initial progress notes between paper charts and a newly implemented electronic health record. J Am Med Inform Assoc 2017; 24 1: 140–4. 27357831 10.1093/jamia/ocw067 PMC7654088

[15] Kaufman DR, Sheehan B, Stetson P, et al Natural language processing-enabled and conventional data capture methods for input to electronic health records: a comparative usability study. JMIR Med Inform 2016; 4 4: e35. 27793791 10.2196/medinform.5544 PMC5106560

[16] Gellert GA, Ramirea R, Webster SL. The rise of the medical scribe industry: Implications for the advancement of electronic health records. JAMA 2014; doi: 10.1001/jama.2014.17128. 10.1001/jama.2014.17128 25504341

[17] Payne TH, Perkins M, Kalus R, et al The transition to electronic documentation on a teaching hospital medical service. AMIA Annu Symp Proc 2006: 629–33. 17238417 PMC1839294

[18] Payne TH, Alonso WD, Markiel JA, et al Using voice to create hospital progress notes: description of a mobile application and supporting system integrated with a commercial electronic health record. J Biomed Inform 2018: 93–96. 10.1016/j.jbi.2017.12.004 PMC12974612 29233669

[19] Canada Health Infoway System And Use Assessment Survey. http://healthit.ahrq.gov/health-it-tools-and-resources/health-it-survey-compendium (Accessed January 25, 2014).

[20] Stetson PD, Bakken S, Wrenn JO, et al Assessing electronic note quality using the Physician Documentation Quality Instrument (PDQI-9). Appl Clin Inform 2012; 03 02: 164–74. 10.4338/ACI-2011-11-RA-0070 PMC3347480 22577483

[21] Neamatullah I, Douglass M, Lehman LH, et al Automated de-identification of free-text medical records. BMC Med Inform Decis Mak 2008; 8 1: 32. 18652655 10.1186/1472-6947-8-32 PMC2526997

[22] Sackett DL, Richardson WS, Rosenberg W, Haynes RB. Evidence-Based Medicine. How to Practice and Teach EBM. New York, NY: Churchill Livingston Inc; 1997.

[23] HL7. Introducing HL7 FHIR. http://www.hl7.org/implement/standards/fhir/summary.html (Accessed March 9, 2016).

[24] Mandl KD, Kohane IS. A 21st-century health IT system—creating a real-world information economy. N Engl J Med 2017; 376 20: 1905–7. 28514623 10.1056/NEJMp1700235

[25] Payne TH, tenBroek AE, Fletcher GS, et al Transition from paper to electronic inpatient physician notes. J Am Med Inform Assoc 2010; 17 1: 108–11. 20064811 10.1197/jamia.M3173 PMC2995621

[26] Solti I, Aaronson B, Fletcher F, et al Building an automated problem list based on natural language processing: Lessons learned in the early phase of development. AMIA Annu Symp Proc 2008: 687–91. 18999050 PMC2655946

[27] Payne TH, Patel R, Beahan S, Zehner J. The physical attractiveness of electronic physician notes. AMIA Annu Symp Proc 2010; 2010: 622–6. 21347053 PMC3041462

